# Supplementary material for: Are carbon emissions trading and green financial instruments synergistic? -Comprehensive quantitative research based on content analysis
Source: PLoS One. 2024 Mar 7;19(3):e0298601. doi: 10.1371/journal.pone.0298601 (PMC10919601; doi:10.1371/journal.pone.0298601)
Supplement: S1 Data — (ZIP) [file pone.0298601.s001.zip › Data/地级市Z_F工作报告.zip!/╦─┤¿/─┌╜¡╩╨/└┤╫╘╣1⁄2╓┌║┼í╛┬φ┐╦╩2╛▌═°í┐_2014.docx]

各位代表：

　　我代表市人民政府，向大会报告工作，请予审查，并请市政协委员提出意见。

2013年工作回顾

　　2013年，面对错综复杂的国内外环境，我们在省委、省政府和市委的坚强领导下，在市人大、市政协的监督支持下，坚持以邓小平理论、“三个代表”重要思想、科学发展观为指导，认真学习贯彻党的十八大、十八届三中全会及省委十届三次全会精神，按照市委六届七次全会的总体部署，围绕市六届人大三次会议确定的目标任务，团结奋进、攻坚克难，勤政务实、勇于担当，全市经济平稳较快发展，各项事业全面进步，社会大局和谐稳定，人民群众安居乐业。全年地区生产总值突破1000亿元，达1069.34亿元，增长10.3%；地方公共财政收入37.75亿元，增长22.1%；地方公共财政收入占地区生产总值的比重达3.5%，提高0.37个百分点；税收收入占地方公共财政收入的比重达71.5%，提高3.4个百分点；城镇居民人均可支配收入达21114元，增加1972元，增长10.3%；农民人均纯收入8584元，增加982元，增长12.9%。

　　一年来，我们致力于调整结构、做大产业，发展基础进一步夯实。工业经济提质升位。坚持“工业主导”，规模工业增加值达538.8亿元，增长11.2%；实现利润总额100.04亿元，增长27.7%；五大优势产业实现增加值488.68亿元，占规模工业增加值的比重达90.7%。大力发展高新技术和战略性新兴产业，新增国家高新技术企业8户，新建创新型企业12户；高新技术产业产值占规模工业总产值的比重达11.6%，提高1.6个百分点；战略性新兴产业产值占规模工业总产值的比重达8.9%，提高1.7个百分点。加快建设千亿川威，培育出一批省级以上“小巨人•成长型”中小企业，新增规模以上工业企业28户。完成技改投资200亿元，增长18.6%。关闭、淘汰24户落后产能企业。强化品牌建设，新获四川著名商标7枚，17个争创四川名牌产品通过现场审查。内江经开区成功升级为国家级经济技术开发区，与成渝经济区（内江）钒钛工业园、隆昌经济开发区一并列入全省重点产业园区“51025”发展计划；七大产业园区拓展承载面积6.75平方公里，新入驻单项投资5000万元以上的企业（项目）53个。农业农村经济稳中有升。粮食产量151.22万吨，增加4.78万吨，增长3.26%。现代农业加快发展，累计建成农业、林业、畜牧、水产等现代示范园区22个、万亩现代农业示范区15个，新建粮经复合型农业产业基地7.51万亩，建成高标准农田8.6万亩；新通过认证无公害农产品12个、国家地理标志保护农产品1个、绿色食品1个；新增市级龙头企业5家、省级示范农民合作组织13个，农业产业化经营带动农户面达70%以上。新农村建设加快推进，新建新村聚居点148个、新农村综合体5个。农村生产生活条件明显改善，启动兴水惠民“五年行动计划”，治理水土流失面积148.03平方公里，小型病险水库除险加固“三年攻坚战”任务圆满完成，威远县象鼻咀水库完成主体工程，东兴区联合水库完成开工准备；新建农村公路1148.59公里；改造农村土坯房4.34万户；增加农村沼气用户1.03万户。第三产业提档升级。第三产业增加值实现231.69亿元，增长10.7%，超过地区生产总值增速；新增限额以上商贸流通企业287户，实现社会消费品零售总额316.12亿元，增长14.5%。北京华联内江购物中心投入运营，万达广场、内江国际家居商贸城、资中东林坝综合体、威远雄飞·国际新城、隆昌都英·新世纪广场等项目加快实施。提升改造特色街区，江华街、东兴老街等一批特色街区建成投运。新建和改造提升城区标准化农贸市场7个。开展“为民惠民”等各类促销活动29项，累计实现直接交易额逾10亿元。全市旅游总收入实现110.29亿元，增长28.0%。金融机构本外币各项存款余额1079.47亿元,比年初增加189.71亿元，增长21.3%；贷款余额514.35亿元，比年初增加107.31亿元，增长26.4%。

　　一年来，我们致力于规划引领、扩容提质，发展空间进一步拓展。坚持中心带动、组团互动、梯次联动，多措并举加快新型城镇化进程，全市新增建成区面积16.1平方公里，城镇化率达42.67%。注重推进规划编制。累计投入1.2亿元，完成内江城市总规修编和13项专项规划、8项控规、4项城市设计的编制工作，内江城区和县城控规覆盖率分别达95%和75%。加快推进内江新城建设。新开工项目59个，续建54个，竣工28个，完成投资142.8亿元。汉安大道东延线、西林大道北延线等9条道路竣工，东城路、汉安大道西延线等加快推进，中心组团道路骨架基本形成。新城20.8万平方米安置还房完成主体工程。同步推进旧城改造。实施旧城改造项目47个，完成投资9.3亿元，汉安大道、大千路、翔龙路等27条主次干道改造基本完成。扎实推进城市创建，加强市容环境卫生专项治理，荣获“省级园林城市”、“四川省城乡环境综合治理环境优美示范城市”称号，第三届省级文明城市通过验收。合力推进县城新区和重点新镇建设。资中县城新增建成区1.5平方公里，威远县城建成区突破20平方公里，隆昌县新城核心区路网骨架基本形成；全国发展改革试点镇——连界镇加快建设，白马、界市、球溪、银山被列入全省第一批“百镇建设试点”镇。

　　一年来，我们致力于深化改革、扩大开放，发展活力进一步释放。各项改革整体推进。以农村产权制度改革为核心的统筹城乡综合配套改革试点取得新成效，完成24个乡镇、406个村、4294个村民小组的农村产权制度改革任务，探索出“三自一引”、“五步工作法”和“三化”模式、农业“BOT”模式等全省领先的经验做法，流转土地1.28万亩，带动当地农民人均增收3000元。放宽城镇落户条件，新增非农业户籍人口1.8万人。加强农村金融改革，扩大林权抵押贷款，域内林权抵押贷款2685万元。实施城乡养老保险并轨。顺利开展“营改增”试点。完成食品药品监管系统机构改革、公共资源交易服务中心组建、市区城市管理体制调整、内江经开区管理体制调整等工作。事业单位分类工作基本完成。开放合作成效显著。充分利用省上举办的“三大投资促进活动”和“全球华商大会”、“成都财富论坛”、“第十四届西博会”等招商平台，主动打好引进央企、外企、民企“三大战役”，新引进大型央企项目25个、知名外企项目12个、民企500强项目12个。全市实际到位市外资金548.25亿元，同口径增长35.5%。全年外贸进出口总额3.63亿美元，增长15.7%。

　　一年来，我们致力于稳定投资、狠抓项目，发展后劲进一步增强。项目投资取得突破。深入开展“项目突破年”活动，全年竣工项目709个，开工500万元以上项目806个，全社会固定资产投资完成575.93亿元，增长25.1%。狠抓要素保障，促进项目建设，全年获批土地突破1000公顷，达1143.2公顷，同比增加148.2公顷；争取中央和省项目资金118.47亿元，增长7.33%；实现平台融资51亿元，内投集团发行18亿元企业债券获国家发改委批准。交通建设扎实推进。围绕“30分钟内江”目标，加快建设区域性综合交通枢纽。成渝客专内江段线下工程基本完成，内江北站、资中北站、隆昌北站站房建设全面启动，连乐铁路前期工作扎实推进；内威荣高速公路、自隆高速公路加快建设，新入城线玉王庙互通立交建设顺利推进，花园滩大桥开工建设，城市过境高速公路、内江综合客运中心站完成前期准备工作。

　　一年来，我们致力于改善民生、促进和谐，发展成果进一步惠民。“十大民生工程”任务全面完成。全市民生支出达109.49亿元，占公共财政支出的68.89%。就业创业工作继续加强，对转移农村劳动力实行实名登记，新增城镇就业人数4.88万人，城镇登记失业率3.66%。社会保障体系不断完善，五大保险参保人数达325.42万人次。全市企业用工最低工资标准上调至1070元/月。扶贫救助力度继续加大，城乡居民最低生活保障标准分别提高至320元/月·人和1920元/年·人。新改扩建敬老院10所,“五保”老人集中供养率达45%。开工建设保障性住房6471套，改造（正在建设）棚户区7135套，为农民工提供公共租赁住房2238套。加强市场监管，保持物价基本稳定，居民消费价格总指数上涨2.3%，控制在调控目标之内。各项事业全面发展。加快发展教育事业，学前三年毛入园率达87.15%，义务教育均衡发展得到加强，普通高中教育稳步发展，内江职院顺利迁入新校区，隆昌幼师创建高等专科学校通过省高评委评估。自主创新能力有所提升，申请专利472项，新建市级院士（专家）工作站4个。群众性文化活动更加丰富，建成省级示范乡镇综合文化站13个；启动农村地面无线数字电视工程建设，建成市、县级服务平台和信号覆盖网络，发展用户1.6万户；新增通宽带行政村146个。群众体育活动蓬勃开展，“周末快乐体育”成为四川全民健身品牌，内江籍运动员在国际、国内赛事中再获佳绩。医疗服务体系不断健全，市中医院二期工程等项目进展顺利，市第一人民医院新区医院、市第二人民医院儿童医疗服务体系等项目前期工作有序推进。低生育水平持续稳定，人口自然增长率为4.91‰。生态环境得到改善。强化饮用水源和甜城湖保护管理，城市生活污水处理率85%，生活垃圾无害化处理率94%，解决了23.67万农村人口饮水安全问题。强化环境约束和节能减排，顺利完成2013年主要污染物总量减排任务，万元地区生产总值能耗下降3.06%，沱江干流内江段水质全年达到Ⅲ类及以上标准，城区空气质量达到和好于国家二级以上的天数314天，全市森林覆盖率31.85%。社会保持安全稳定。狠抓安全生产工作，隐患排查、打非治违、煤矿清理整顿和兼并重组取得阶段性成果；成功应对4次沱江洪峰过境；强化“安全社区”和“安全生产标准化”企业建设，安全生产形势持续稳定好转，全市各类生产经营性伤亡事故、死亡人数、受伤人数分别下降16.95%、8.11%、27.01%。加强食品药品监管，全市未发生较大及以上食品安全事故。深化平安内江建设，健全社会矛盾调解体系，深入开展“大排查、大调解”专项行动，来信来访下降15.6%；公安派出所正规化建设和公安基层基础建设取得新成效，公众安全感满意率达到95.4%。应急处突能力进一步提升，驰援芦山抗震救灾迅速有效，各类突发事件得到及时妥善处置。

　　国防后备力量建设扎实推进，正规化建设水平全面提高，新兵征集任务圆满完成，再创省级双拥模范城进入迎检阶段。第三次全国经济普查全面开展，统计调查、气象、人防、外事侨务、民族宗教等工作扎实推进，机构编制、档案、保密、史志、无线电、红十字、老龄、妇女儿童和残疾人等事业取得新成绩。

　　一年来，我们致力于转变作风、提升效能，政府自身建设进一步加强。认真贯彻执行中央、省、市关于改进工作作风、密切联系群众各项规定，切实查纠“四风”问题，厉行勤俭节约，转变文风会风，整治“庸懒散奢”，服务意识进一步增强。自觉接受市人大及其常委会的法律监督、工作监督和市政协的民主监督。广泛听取各民主党派、工商联、无党派代表人士和人民团体的意见。实行市长、全体副市长亲自领办一件以上重点建议、提案制度和提案协商办理制度，共办理人大代表议案1件、建议和批评意见124件，办理政协委员提案275件，办结率100%，满意率98%以上。深入推进法制宣传教育和依法治理工作，“六五”普法和法治城市、法治县（区）创建工作全面开展。大力实施简政放权，全面清理规范行政审批事项，保留市级行政审批项目85项，审批项目平均提速78%。加快行政权力依法规范公开运行平台建设，基本实现市、县、乡三级行政效能电子视频监察系统全覆盖。创新开展“千企万人”综合测评活动，查办各类行政效能投诉746件。加强对政府性投资、招投标、民生工程及财政专项资金的监管，完成政府投资工程预决算审计项目618个。严格落实党风廉政建设责任制，不断完善惩治和预防腐败体系，进一步强化对决策执行、民生保障、廉政勤政的监督，政纪立案55件，政纪处分55人。

　　各位代表，2013年，全市主要经济指标增速均高于全省平均水平，总量在全省的排位实现提升或保持上年水平，发展质量持续提升，社会局面持续稳定，人民生活持续改善。成绩来之不易！在此，我谨代表市人民政府，向430万内江人民，向人大代表、政协委员、各民主党派、工商联、无党派人士、各人民团体，向驻内部队、武警官兵、政法干警，向关心、支持和参与内江建设发展的各界朋友，表示衷心的感谢并致以崇高的敬意！

　　2013年，受宏观经济下行、结构调整升级和市场消费需求不足等因素影响，全市地区生产总值、服务业增加值、规模工业增加值、社会消费品零售总额、城镇居民人均可支配收入、农民人均纯收入等6项指标增速虽超过全省平均水平，但未达到我市年初确定的预期目标。同时，我市经济社会发展过程中还存在一些不容忽视的困难和问题，主要体现在：三次产业结构不协调，服务业占比明显偏低，高新技术产业和战略性新兴产业比重偏低，产业转型升级还任重道远；投资结构不优，产业投资增长较慢；财政收入占经济总量比重较低，税源培植和融资平台建设有待加强；城镇化水平较低，城镇配套设施还不完善，城区交通拥堵现象突出；生态环境、教育、文化、卫生、住房、收入分配等方面，与群众期望还有较大差距；部门“中梗阻”现象时有发生，政府作风效能建设还需深化。对这些困难和问题，我们将高度重视，积极采取针对性措施，认真加以解决。

2014年主要工作任务

　　2014年是全面深化改革的第一年，也是实施“十二五”规划的关键一年。当前，发展已进入经济增长速度换档期、结构调整阵痛期和前期刺激政策消化期，既要抓住机遇保持经济合理增长，又要抓住机遇推进经济结构调整；全面深化改革进入“深水区”和攻坚期，既要以破冰的勇气全面推进，又要以高度的智慧保持平稳。尤其面对省委对川南经济区“两个率先”和内江要在“五个方面”实现新突破的要求，面对“底子薄、不协调、欠发达”的基本市情，在当前以及今后的工作中，我们既要坚持用改革统领经济社会发展全局，又要以创新激发经济社会发展活力，不断增强内江发展的内生动力；既要坚持加快发展这个第一要务，又要把转型升级作为主攻方向，努力实现更高水平、更好质量、更加协调、更可持续的发展；既要坚持将改善民生作为第一追求，又要牢记维护安全稳定的第一责任，主动顺应群众的期待、全力增进人民福祉。

　　2014年政府工作的总体要求是：认真贯彻落实党的十八大、十八届三中全会和省委十届三次、四次全会精神，按照市委六届八次全会的统一部署，用改革统领经济社会发展全局，坚持科学发展、加快发展的工作指导思想，深入实施“两化”互动城乡统筹、创新驱动、改革开放“三大战略”，加快推进新型工业化、新型城镇化和农业现代化，着力保障和改善民生，不断提高经济发展质量和效益，加快建设充满希望、充满活力的幸福美丽内江。

　　2014年主要预期目标建议：地区生产总值增长9.5%，其中第一产业增加值增长3.5%、第二产业增加值增长10.5%（规模工业增加值增长10.2%）、第三产业增加值增长10.6%；地方公共财政收入增长16.0%；社会消费品零售总额增长13.5%；全社会固定资产投资增长16.0%；城镇居民人均可支配收入增长10.0%，农民人均纯收入增长11.0%；化学需氧量、氨氮、二氧化硫、氮氧化物排放量控制在省下达的目标以内，万元地区生产总值能耗同比下降2.0%；居民消费价格涨幅控制在3.5%左右；城镇登记失业率控制在4.0%以内；人口自然增长率控制在5.6‰以内。

　　围绕上述总体要求和预期目标，重点抓好以下工作：

　　一、突出创新驱动，进一步推动产业转型升级

　　加快建设高新技术产业园。积极创建国家级高新技术产业园，抓紧做好园区规划，优先保障用地需要，加快推进基础设施建设，积极培育引进电子信息、生物制药、高端装备制造、节能环保等产业，加快引进一批研发机构。

　　进一步提升自主创新能力。全面推进高新技术产业发展、科技成果转化、创新体系培育、产学研用协同创新“四大工程”，以科技创新引领和支撑产业升级，力争高新技术产业、战略性新兴产业产值占规模工业总产值的比重分别提高2.5个百分点以上。加大地方公共财政对科技的投入，加快创建国家级科技企业孵化器，新增科技孵化园面积1万平方米以上，新培育国家高新技术企业21户。深化产学研合作，鼓励重点骨干企业联合高等院校、科研单位建立产学研技术联盟或产业技术研究院，新建省级工程技术研究中心1个、院士（专家）工作站2个，组织实施重大科技成果转化项目30项。加强知识产权保护，新增专利申请550件，增长15%。积极培育品牌建设示范区和质量强市（镇）示范城（镇），新培育中国驰名商标1枚、四川省著名商标4枚、四川名牌产品10个。加大对创新人才的培养、引进，激发其创新活力。

　　加快老工业基地调整改造。完成内江老工业城市调整改造规划编制，落实“腾笼换鸟”政策措施，启动实施马鞍山、麻柳坝老工业区企业搬迁改造，推动企业搬迁入园、转型升级、集聚集群发展。用新技术、新装备改造提升机械汽配、冶金建材、医药化工等传统优势产业。加快建设中铁建重工集团西南产业园、中建材节能玻璃产业园等重大技改项目，全年投入技改资金200亿元以上。做好千亿川威培育工作，发展壮大一批100亿元企业、50亿元企业，力争全年新增规模工业企业20户、5亿元企业7户、10亿元企业5户。全力实施“51025”重点园区计划，完成重点园区总规编制，建成600亿元园区1个、300亿元园区1个、200亿元园区2个，力争七大园区新增承载面积5平方公里，新入驻单项投资5000万元以上企业（项目）30个。

　　加快发展现代服务业。把加快发展现代服务业作为转型升级的战略重点，力促服务业发展提速、比重提高、水平提升，力争服务业增加值增速不低于第二产业增加值增速。认真实施《市人大常委会关于加快内江商贸发展的决议》，在完善规划、深化改革创新、落实扶持措施、加强目标考核上下功夫，提高市场集中度、扩大商贸辐射力、增强龙头带动力。推进城市综合体建设，提升特色商业街区同业度，加快建设万达广场等一批城市综合体，夯实现代服务业发展基础。大力发展电子商务和现代物流，实施电子商务提速工程，启动建设电子商务城，积极发展网购网销等新型商业模式；引导渤商西部交易中心增加交易品种，尽快启动再生资源交易平台建设。积极培育第三方物流，积极发展智慧物流，加快白马现代物流园、苏宁川南配送中心、资中科伦医贸物流、威远连界钢铁物流、隆昌粮食物流中心建设。大力培育发展现代旅游业和会展经济，实施“一三八”计划，着力打造“文化之旅、山水之旅、美食之旅”三大旅游品牌，旅游总收入增长20%以上；加快内江国际会展中心建设，适时举办各类展会。加快现代金融业发展，规划建设金融一条街，积极引进股份制商业银行，稳妥发展一批新型金融机构，积极鼓励金融创新，做大做优区域融资规模，鼓励和支持企业拓宽融资渠道。

　　加快现代金融业发展，规划建设金融一条街，积极引进一批新型金融配套机构，鼓励和支持企业拓展融资渠道。

　　二、高度重视“三农”工作，加快建设幸福美丽新农村

　　着力发展现代农业。抓好“米袋子”、“菜篮子”工程，在确保粮食安全的基础上，继续推进现代畜牧业试点市和现代农业、林业、畜牧业重点县项目建设，新建粮经复合现代农业产业基地5万亩、现代农业产业万亩示范区7个，生猪、家禽规模化养殖比重分别达到64%、48%，新增水产品产量2000吨。抓好资中县银山现代农业园、市中区永安现代农业园、东兴区长江现代农业园、威远县万亩无花果基地建设，提升农业园区发展水平，全力打造国家级农业科技园区。扶持发展新型农业经营主体，鼓励发展农民合作社，按照自愿原则开展家庭农场登记，鼓励发展混合所有制农业产业化龙头企业，加大对新型职业农民和新型农业经营主体领办人的教育培训力度。强化农业科技创新和转化推广，健全农业科技推广服务体系，促进传统农业向现代农业转变。高度重视农产品质量安全，大力发展无公害、绿色、有机农产品，强化农产品地理标志保护，建立可追溯的质量监管体系。

　　改善农村生产生活条件。坚持“小组团、生态化、可持续”发展模式，继续抓好5个新农村综合体建设，因地制宜规划、建设110个新村聚居点和一批幸福美丽新村。继续推进“康庄惠民工程”，新（改）建农村公路300公里以上，力争实现村村通油路（水泥路）目标。协调推进向家坝灌区一期工程建设和长征渠规划修编，开工建设东兴区联合水库，加快资中县两河口水库前期工作。抓好小农水重点县和中小河流防洪治理项目建设，推进石盘滩大型灌区和黄河镇水库中型灌区续建配套与节水改造，新增全域灌溉小区9个。建设高标准农田8万亩，新增农村沼气用户5000户。加强气象灾害防御、动植物疫病防控，提高农业防灾减灾能力。

　　努力促进农民增收。严格落实农民增收负责制，严格执行重农惠农富农各项政策，增加农民转移性收入。提升劳务转移数量和质量，增加农民工资性收入。继续推进农村产权制度改革，完成46个乡镇、676个村、7479个村民小组的确权颁证；健全产权流转服务体系，盘活用好农村产权资源，新增农村土地流转面积3万亩，把“本本”变“本钱”。加强扶贫攻坚和移民扶持，帮助6.31万农村贫困人口脱贫致富。

　　三、以完善规划和健全功能为重点，加快新型城镇化进程

　　科学构建城镇体系。抢抓“优先发展川南城市群”机遇，以新城新区新镇为重点，坚持规划引领，注重适度超前、多规衔接，加快形成以百万人口特大城市为中心、中等城市为骨干、小城镇为基础的现代城镇体系。加快内江新城建设，完成内江市新一轮城市总规上报、谢家河片区北部拓展区控规编制和椑木组团城市设计；拓展内江新城路网骨架体系，完成清溪路、玉屏街、甜城大道北延线、北环线一二段、东城路等骨干道路建设。加快县城新区建设，强化对县城的规划指导，完成隆昌县县城总规修编，继续编制三县县城控规和重要专项规划，确保县城控规覆盖率达85%；支持三县县城新区扩容提质，支持两区改旧城、拓新区，加快建设城南新区、白马组团和椑木组团。加快重点小城镇建设，科学制定小城镇规划，编制完成5个镇乡的统筹城乡全域规划、30个镇乡总体规划、10个镇控规；重点抓好纳入全省“百镇建设试点行动”的乡镇建设，增强小城镇的承载能力和服务功能。

　　逐步完善城镇配套。针对城市新区加快建设和城市人口快速增长的实际，在拓展路网的基础上，坚持把完善城镇配套作为当前城镇建设的重要任务。加快文化、体育、医疗、教育、养老、环卫及综合农贸市场、社区活动场所、CNG加气站等公共服务配套设施建设，加快广场、公园、生态绿地建设，抓紧完善综合交通、供排水和水系统、绿地系统等专项规划，不断增强城镇的宜居宜业度。加快桐梓坝小学、市一院新区医院建设，推动市新体育中心、市文化中心、六中实验学校、五星小学等项目尽快开工，启动内江师院、内江医科学校整体迁建和市技工校、一职中、六职中“三校合一”工程；完成长江森林公园、大梁山风景区、小青龙滨河公园、塔山公园规划，开工建设五星水库湿地公园、牛毛山公园。围绕完善城镇功能配套，推进城中村、背街小巷、商业中心改造提升工程，强力推进城市棚户区改造，加快实施老旧院落综合整治。强力推进城区交通治堵三年攻坚战，以改造城区交通堵点、完善道路交通管理设施、健全路网和桥梁体系、加强停车场所规划建设、优化公交线路和过境交通组织等为重点，综合治理城区交通拥堵。

　　加强城市管理。完善市、区两级城市管理体制，建立市、区、街道、社区“四级联动”的城管长效机制。深化城乡环境综合治理，加大城市综合执法力度，对乱停乱放、占道经营、油烟、噪声、扬尘、建筑垃圾等实施专项治理，积极推进国家卫生城市、国家园林城市创建工作。加快推进智慧城市建设，重点在城建、城管、交通、医疗、天网、电力、行政权力公开运行等领域推进智能化管理。

　　加快交通枢纽建设。围绕将内江打造成“成渝立交桥”目标，着力完善综合交通运输网络，启动城市过境高速公路、黄荆坝大桥、邓家坝大桥、内江综合客运中心、公交枢纽中心站、连乐铁路建设；加快建设成渝客运专线内江段及站房、内威荣高速公路（内江段）、自隆高速公路（内江段）、花园滩大桥；完成新入城线玉王庙互通立交建设；做好内泸城际铁路、绵遂内自宜城际铁路以及内江至资中、内江至隆昌、内江至威远3条快速通道等项目前期工作。按照“顶层设计、规划先行、重点突破、勇于创新”的要求，抓紧启动内江城市轨道交通线网规划和建设规划编制工作，力争2014年底前完成第一条城市轨道交通线路项目前期工作。

　　四、优化投资结构，加大投资力度，扩大消费需求

　　保持投资增势。着力扩大民间投资，鼓励民间资金进入农业农村、基础产业、基础设施、市政公用设施、社会事业、金融服务等领域。稳定政府性投资增长，重点抓好产业、民生、基础设施、节能环保等领域投资，力争产业投资占固定资产投资的比重提高1个百分点以上。全年计划安排270个市级重点建设项目，年度计划投资318亿元。积极对接中央和省中长期规划，论证、包装、申报一批重大项目，充实项目储备库。加大土地整理和收储工作力度，集中集约利用土地，提高土地利用效益。

　　扩大城乡消费。优化商业网点布局，加快打造谢家河、高铁新城、邱家嘴等现代商圈。打造一批名特优产品专卖店，做响一批特色餐饮品牌，扩大内江消费辐射力。保持汽车、房地产等大宗消费平稳健康发展。积极培育信息消费、健康养老、休闲旅游、文化娱乐、家政服务等消费热点。搭建各类促销平台，开展“惠民购物行动”，繁荣假日消费市场。进一步完善流通体系，加强产销对接和农超对接，推进农村商业连锁经营和统一配送，活跃农村消费市场。加强产品质量监管，严厉打击假冒伪劣产品，创造良好消费环境。

　　五、全面深化改革开放，着力增强发展动力和活力

　　继续推进改革。推动国有企业完善现代企业制度，积极发展混合所有制经济。放宽市场准入条件，促进民营经济发展。加强市场监管，引导中介组织强化行业自律，充分激发各类市场主体活力。完善加快县（区）发展振兴的措施，促进县域经济提质升位。稳妥推进资源性产品价格改革，推动建立天然气市场化定价机制，调整优化销售水价结构。深化财税体制改革，完善市以下分税制财政体制。健全政府债务预警机制，加强政府债务管理控制。深化城乡社会保障制度改革，建立城乡“一个体系、两个险种、多个档次”的养老保险和“一个制度、多档选择、同步补充”的医疗保险体系，实施城乡养老保险并轨、转换接续政策，将进城落户农民纳入城镇住房和社会保障体系。深化户籍制度改革，建立财政转移支付同农业转移人口市民化挂钩机制。启动实施一方是独生子女的夫妇可生育两个孩子的政策；进一步推进中小学教育质量评价综合改革，深化义务教育招生制度改革；加快经营性文化单位转企改制。

　　深化开放合作。全力打好“高新产业引进、知名企业引进、人才带项目引进”三大攻坚战，全年到位市外资金同口径增长15%以上。围绕高端制造业和现代服务业，瞄准世界500强和国内知名企业，引进一批科技含量高、产业关联度强、就业贡献大的产业项目，确保到位资金中制造业项目资金占比达到60%。把招才引智与招商引资结合起来，突出“优质稀缺”，着力引进一批带着项目的高层次人才。抢抓国家扩大内陆沿边开放和建设长江经济带、丝绸之路经济带以及全省实施多点多极支撑发展战略等机遇，加快建设川渝合作“桥头堡”。进一步加强厅市合作、校地合作和民间交流合作，促进互利发展。积极拓展海外市场，抓好外贸基地建设，扶持有优势有潜力的企业发展对外贸易。

　　六、加强生态建设与保护，积极建设资源节约型和环境友好型社会

　　狠抓节能减排。积极发展节能产业，坚决关闭和淘汰落后产能。扎实抓好重点领域节能，大力发展循环经济，鼓励节能减排技术开发与推广应用，实施一批余热余压利用、绿色照明等节能改造工程。推进城市能源计量中心（内江）建设，全面实行节能评估和审查制度，实行能源利用状况报告制度，推进能耗在线监测工作。严控主要污染物排放，严把环境准入条件，确保不发生重特大环境污染事故。加强空气质量监测，启动PM2.5监测及信息发布工作。建立完善机动车尾气检测体系，全面实现机动车环保标志管理，加快淘汰高排放车辆。

　　强化水资源保护。严格落实水资源保护、管理制度，强化用水总量、用水效率和水功能区纳污“三条红线”管理。在城镇集中式饮用水源保护区，深入开展工业污染、城市污染、农村面源污染治理，加强水质常规监测和应急监测，确保集中式饮用水源水质安全监测全覆盖，提高水质达标率。继续实施主要河流水污染防治，突出抓好威远河、隆昌河治理。完成内江第一污水处理厂排污口下移工作，加快推进内江第三水厂及第二污水处理厂建设，确保城区污水集中处理率、生活垃圾无害化处理率分别提高2个百分点以上。

　　加强生态建设。落实主体功能区规划，划定生态保护红线，推进东兴区全国水土流失治理试点县、资中县全国生态文明示范工程试点县、威远县全国石漠化综合治理重点县建设。加强农村人居环境综合整治，创建生态乡镇7个、生态村10个、生态家园100户。大力开展植树造林活动，进一步巩固退耕还林、天然林保护和城周绿化、通道绿化、新村绿化工程成果，确保森林覆盖率提高0.6个百分点。

　　七、切实办好民生实事，全面发展各项事业

　　全力保障和改善民生。继续推进就业促进、扶贫解困、民族地区扶贫、教育助学、社会保障、医疗卫生、百姓安居、民生基础设施、生态环境、文化体育等“十大民生工程”，确保今年民生支出占公共财政支出比重稳步提升。突出抓好20件民生实事：实现义务教育全免费，对全市义务教育阶段学生作业本费应免尽免；免除全市1.65万名家庭经济困难高中学生学费；加大城乡公办幼儿园建设力度，减免1.05万名家庭经济困难在园幼儿保教费；全面取消县级公立医院药品加成；全面实行婚前免费体检；提高城乡居民医疗保险水平，财政补助标准达到320元/年•人；提高城乡居民养老保险水平，基础养老金标准达到60元/月•人；新增、改造5020张养老床位；建设78个城乡社区日间照料中心，为5.06万名困难家庭的失能老人和80周岁以上的高龄老人提供居家养老服务；对80岁以上老人给予不低于120元/年•人的高龄补贴；资助3.31万名重度残疾人护理费用；对初次创业并吸纳3名以上劳动者就业的高校毕业生给予1万元补贴；继续实施农村危房改造；启动建设保障性住房5826套，改造危旧房和棚户区13217户；续建安置还房162.35万平方米，年内完成投资8.63亿元，累计投资进度达到70%以上；将30%公租房定向出租给农民工；解决13.54万农村人口和2.6万学校师生饮水安全问题；改善乡村公路75公里；建设320公里公路安全护栏；实施899个村公共服务设施运行维护补助。

　　全面推进社会事业发展。积极创建省级创业型城市，完善城乡一体的就业失业登记制度，实现城乡公共就业服务全覆盖，新增城镇就业3.7万人。适时上调全市最低工资标准。深入推进素质教育，加快推进城乡义务教育均衡发展进程，大力发展学前教育，稳定高中教育发展规模，构建产教融合的现代职业教育体系，推动高等院校内涵式发展。加强基层医疗卫生机构标准化建设，提高疾病防控和卫生应急处置能力。广泛开展群众性文化创建活动，推进5个“幸福美丽新村(社区)文化院坝”试点，加快城乡有线广播电视网络综合信息化建设，加强非物质文化遗产保护工作。深入实施全民健身计划，积极开展群众性体育活动，做好省第十二届运动会训练备战。

　　抓好民兵应急分队和预备役部队建设，全面普及国防教育，不断提升国防动员和后备力量建设质量。继续做好人口和计划生育工作，稳定低生育水平。依法管理民族宗教事务。完成第三次全国经济普查工作。启动全市应急广播体系建设。推动统计调查、新闻出版、无线电、气象、水文、检验检疫、保密、档案、史志、人防、老龄、妇女儿童、残疾人、红十字、外事侨务等事业协调发展。

　　八、坚决维护安全稳定，努力促进社会和谐

　　加强安全生产。牢固树立“安全发展”理念，严格落实安全生产责任制和安全生产倒查倒逼工作机制。开展煤矿和非煤矿山专项整治活动；加强道路交通安保设施建设，开展道路交通安全综合整治攻坚年行动；继续加强消防、烟花爆竹、危化物品、建筑施工、食品药品安全等重点行业领域监管。强化安全生产教育培训，推进安全社区建设，夯实安全生产基层基础。推进安全隐患排查治理体系建设，构建安全隐患排查治理常态化机制。建立市、县、企业三级安全生产监测监控系统，完善安全应急救援体系，有效防范和坚决遏制重特大生产安全事故。

　　维护社会稳定。积极探索建立更加科学完善的社会治理体系，不断提高社会治理水平。坚持源头治理，推进城乡社区建设，健全基层综合服务管理平台。扩大社会参与，加大社会组织培育引导力度，支持企事业单位、人民团体在社会治理中发挥更大作用，丰富居民参与社会治理的渠道和形式。创新有效预防和化解社会矛盾体制，健全重大决策社会稳定风险评估机制，推动“大调解”体系向纵深延伸。深化平安内江建设，全面推进网格化服务管理，加强公安“三化”建设，提升“六种能力”，加快构建立体化社会治安防控体系，严厉打击各类违法犯罪行为，确保全域平安。进一步健全突发事件应急处置机制，提高各类突发事件应急处置能力。

　　九、加强政府自身建设，努力建设人民满意政府

　　严格依法行政。牢固树立法治意识，全面贯彻落实《四川省依法治省纲要》，大力开展法制宣传教育和依法治理工作，积极推进法治城市、法治县（区）创建工作，引导公务人员更好地运用法治思维和法治方式履职行权。规范行政处罚裁量权，抓好行政复议工作，加强重点领域市场监管，保障人民合法权益。自觉接受市人大及其常委会的法律监督、工作监督和市政协的民主监督，高度重视司法监督、舆论监督和人民群众监督，认真办理人大代表议案、建议批评意见和政协委员提案，广泛听取民主党派、工商联、无党派人士和人民团体的意见。积极支持工会、共青团、妇联等群团组织开展工作。完善决策机制，广泛开展专家论证、技术咨询、决策评估、公示听证，推进决策的科学化、民主化。全面推行政务公开，加快电子政务建设，增强政府工作的透明度。加快政府职能转变，完成市县政府部门机构改革工作，完善事业单位分类改革配套政策，稳步推进行政管理体制改革。取消和下放部分市级行政审批事项，清理规范非行政审批事项，提升政务服务水平。

　　改进工作作风。严格落实中央、省、市关于改进工作作风、密切联系群众各项规定，按照“照镜子、正衣冠、洗洗澡、治治病”的总要求，扎实开展好群众路线教育实践活动，着力解决“四风”突出问题，解决关系群众切身利益的问题，解决联系服务群众“最后一公里”的问题。加强节约型政府建设，严格执行厉行节约、反对奢侈浪费的规定，压缩一般性财政支出，严格控制“三公”经费，降低行政运行成本。深化“千企万人”综合测评活动，加大对不作为、慢作为、乱作为的惩治力度，全面提升行政效能，进一步优化政务服务环境。

　　抓好廉政建设。严格落实党风廉政建设责任制，深化源头治理，不断完善惩治和预防腐败体系。规范财政预算，加强审计监督、行政监察和政务督查，扩大政府性投资项目评审覆盖面，堵住公共资源交易、公共资金管理等腐败易发多发领域的漏洞。严肃查办商业贿赂和严重侵害群众利益案件，严肃查办群体性事件和重大责任事故背后的腐败案件。

　　各位代表，让我们紧密团结在以习近平同志为总书记的党中央周围，按照省委省政府的决策部署，在市委的坚强领导下，在市人大、市政协的监督支持下，风正心齐，务实重干，砥砺奋进，攻坚克难，为开创内江科学发展、加快发展的崭新局面，为建设充满希望、充满活力的幸福美丽内江而努力奋斗！
